# {5,5′-Dimeth­oxy-2,2′-[4,5-dimethyl-*o*-phenyl­enebis(nitrilo­methyl­idyne)]diphenolato}nickel(II)

**DOI:** 10.1107/S1600536810051834

**Published:** 2010-12-15

**Authors:** Atefeh Sahraei, Hadi Kargar, Reza Kia, Muhammad Nawaz Tahir

**Affiliations:** a Department of Chemistry, School of Science, Payame Noor University (PNU), Ardakan, Yazd, Iran; b Department of Chemistry, Science and Research Branch, Islamic Azad University, Tehran, Iran; c X-ray Crystallography Laboratory, Plasma Physics Research Center, Science and Research Branch, Islamic Azad University, Tehran, Iran; d Department of Physics, University of Sargodha, Punjab, Pakistan

## Abstract

In the title Schiff base complex, [Ni(C_24_H_22_N_2_O_4_)], the Ni^II^ atom shows a square-planar geometry. The dihedral angles between the central benzene ring and the two outer rings are 4.79 (15) and 7.54 (15)°. In the crystal, mol­ecules are connected through inter­molecular C—H⋯O hydrogen bond, resulting in chains extending along the *c* axis. The crystal structure is further stabilized by inter­molecular π–π inter­actions, with centroid–centroid distances in the range 3.3760 (15)–3.7196 (17) Å.

## Related literature

For background to Schiff base–metal complexes, see: Granovski *et al.* (1993 ▶); Blower *et al.* (1998 ▶). For related structures, see: Elmali *et al.* (2000 ▶); Kargar *et al.* (2010 ▶).
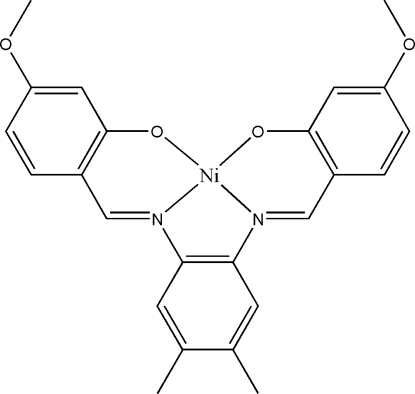


## Experimental

### 

#### Crystal data

[Ni(C_24_H_22_N_2_O_4_)]
*M* *_r_* = 461.15
Monoclinic, 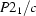

*a* = 11.3244 (10) Å
*b* = 16.5528 (19) Å
*c* = 12.1622 (11) Å
β = 113.261 (6)°
*V* = 2094.5 (4) Å^3^
*Z* = 4
Mo *K*α radiation
μ = 0.96 mm^−1^
*T* = 296 K
0.24 × 0.12 × 0.08 mm

#### Data collection

Stoe IPDS II Image Plate diffractometer
Absorption correction: multi-scan (*MULABS* in *PLATON*; Spek, 2009 ▶) *T*_min_ = 0.872, *T*_max_ = 1.000
13361 measured reflections
4799 independent reflections
3241 reflections with *I* > 2σ(*I*)
*R*_int_ = 0.070

#### Refinement

*R*[*F* ^2^ > 2σ(*F* ^2^)] = 0.048
*wR*(*F* ^2^) = 0.092
*S* = 0.98
4799 reflections
284 parameters
H-atom parameters constrained
Δρ_max_ = 0.25 e Å^−3^
Δρ_min_ = −0.37 e Å^−3^

### 

Data collection: *X-AREA* (Stoe & Cie, 2005 ▶); cell refinement: *X-AREA*; data reduction: *X-AREA*; program(s) used to solve structure: *SHELXS97* (Sheldrick, 2008 ▶); program(s) used to refine structure: *SHELXL97* (Sheldrick, 2008 ▶); molecular graphics: *SHELXTL* (Sheldrick, 2008 ▶); software used to prepare material for publication: *SHELXTL* and *PLATON* (Spek, 2009 ▶).

## Supplementary Material

Crystal structure: contains datablocks global, I. DOI: 10.1107/S1600536810051834/pv2367sup1.cif

Structure factors: contains datablocks I. DOI: 10.1107/S1600536810051834/pv2367Isup2.hkl

Additional supplementary materials:  crystallographic information; 3D view; checkCIF report

## Figures and Tables

**Table 1:** Hydrogen-bond geometry (Å, °)

*D*—H⋯*A*	*D*—H	H⋯*A*	*D*⋯*A*	*D*—H⋯*A*
C7—H7*A*⋯O2^i^	0.93	2.41	3.173 (3)	140

Symmetry code: (i) 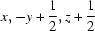.

**Fig. 1.**
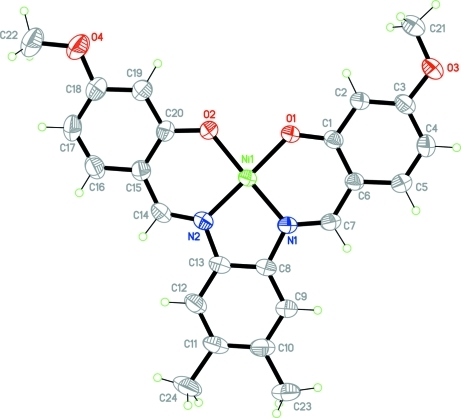
The asymmetric unit of the title compound, showing 40% probability displacement ellipsoids and the atomic numbering.

**Fig. 2.**
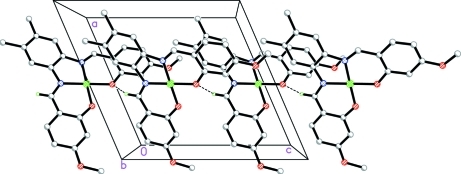
The packing of the title compound viewed down the a-axis showing 1-D infinite chains along the c -axis through the intermolecular C—H···O hydrogen bonds shown as dashed lines; H-atoms not involved in hydrogen bonding were excluded for clarity.

## supplementary crystallographic information

### Comment

Schiff base complexes are one of the most important stereochemical models in transition metal coordination chemistry, with the ease of preparation and structural variations (Granovski et al., 1993). Metal derivatives of the Schiff bases have been studied extensively, and Ni(II) and Cu(II) complexes play a major role in both synthetic and structurel research (Kargar et al., 2010; Elmali et al., 2000; Blower et al., 1998).

In the title compound (Fig. 1), the geometry around the Ni(II) atom is square-planar which is coordinated by O1/O2/N1/N2 donor atoms of the tetradenate Schiff base ligand. The dihedral angles between the central benzene ring (C8–C13), and the two outer rings (C1–C6 and C15–C20) are 4.79 (15) and 7.54 (15)°. The crystal structure is furhter stabilized by intermolecular π–π interactions [Cg1···Cg2^i^ = 3.4737 (17)Å; Cg2···Cg3^i^ = 3.7196 (17)Å; Cg3···Cg3^i^ = 3.3760 (15)Å; Cg1, Cg2, and Cg3 are the centroids of the Ni1/N1/C8/C13/N2, C15–C20, and Ni1/O2/C20/C15/C14/N2 rings, respectively].

### Experimental

The title compound was synthesized by adding bis(4-methoxysalicylidene)-4,5-dimethyl phenylenediamine (2 mmol) to a solution of NiCl_2_. 6 H_2_O (2 mmol) in ethanol (30 ml). The mixture was refluxed with stirring for half an hour. The resultant red solution was filtered. Dark-red plate single crystals of the title compound suitable for *X*-ray structure determination were recrystallized from ethanol by slow evaporation of the solvent at room temperature over several days.

### Refinement

All hydrogen atoms were positioned geometrically with C—H = 0.93-0.96 Å and included in a riding model approximation with U_iso_ (H) = 1.2 or 1.5 U_eq_ (C). A rotating group model was applied to the methyl groups.

### Crystal data

**Table:**

[Ni(C_24_H_22_N_2_O_4_)]	*F*(000) = 960
*M**_r_* = 461.15	*D*_x_ = 1.462 Mg m^−^^3^
Monoclinic, *P*2_1_/*c*	Mo *K*α radiation, λ = 0.71073 Å
Hall symbol: -P 2ybc	Cell parameters from 2525 reflections
*a* = 11.3244 (10) Å	θ = 2.5–29.5°
*b* = 16.5528 (19) Å	µ = 0.96 mm^−^^1^
*c* = 12.1622 (11) Å	*T* = 296 K
β = 113.261 (6)°	Block, red
*V* = 2094.5 (4) Å^3^	0.24 × 0.12 × 0.08 mm
*Z* = 4	

### Data collection

**Table:**

Stoe IPDS II Image Plate diffractometer	4799 independent reflections
Radiation source: fine-focus sealed tube	3241 reflections with *I* > 2σ(*I*)
graphite	*R*_int_ = 0.070
Detector resolution: 0.15 mm pixels mm^-1^	θ_max_ = 27.5°, θ_min_ = 2.0°
ω scans	*h* = −14→14
Absorption correction: multi-scan (*MULABS* in *PLATON*; Spek, 2009)	*k* = −21→20
*T*_min_ = 0.872, *T*_max_ = 1.000	*l* = −15→11
13361 measured reflections	

### Refinement

**Table:**

Refinement on *F*^2^	Primary atom site location: structure-invariant direct methods
Least-squares matrix: full	Secondary atom site location: difference Fourier map
*R*[*F*^2^ > 2σ(*F*^2^)] = 0.048	Hydrogen site location: inferred from neighbouring sites
*wR*(*F*^2^) = 0.092	H-atom parameters constrained
*S* = 0.98	*w* = 1/[σ^2^(*F*_o_^2^) + (0.035*P*)^2^] where *P* = (*F*_o_^2^ + 2*F*_c_^2^)/3
4799 reflections	(Δ/σ)_max_ < 0.001
284 parameters	Δρ_max_ = 0.25 e Å^−^^3^
0 restraints	Δρ_min_ = −0.37 e Å^−^^3^

### Special details

**Table:**

Geometry. All esds (except the esd in the dihedral angle between two l.s. planes) are estimated using the full covariance matrix. The cell esds are taken into account individually in the estimation of esds in distances, angles and torsion angles; correlations between esds in cell parameters are only used when they are defined by crystal symmetry. An approximate (isotropic) treatment of cell esds is used for estimating esds involving l.s. planes.
Refinement. Refinement of F^2^ against ALL reflections. The weighted R-factor wR and goodness of fit S are based on F^2^, conventional R-factors R are based on F, with F set to zero for negative F^2^. The threshold expression of F^2^ > 2sigma(F^2^) is used only for calculating R-factors(gt) etc. and is not relevant to the choice of reflections for refinement. R-factors based on F^2^ are statistically about twice as large as those based on F, and R- factors based on ALL data will be even larger.

### Fractional atomic coordinates and isotropic or equivalent isotropic displacement parameters (Å^2^)

**Table:**

	*x*	*y*	*z*	*U*_iso_*/*U*_eq_	
Ni1	0.52301 (3)	0.15530 (2)	0.57365 (3)	0.03266 (10)	
O1	0.67263 (16)	0.20767 (13)	0.58815 (17)	0.0422 (5)	
O2	0.52566 (17)	0.11985 (13)	0.43077 (16)	0.0418 (5)	
O3	1.05950 (18)	0.35630 (16)	0.7684 (2)	0.0597 (7)	
O4	0.4090 (3)	−0.00753 (17)	0.0642 (2)	0.0735 (8)	
N1	0.51974 (18)	0.19646 (15)	0.7148 (2)	0.0344 (5)	
N2	0.37394 (18)	0.10034 (14)	0.5568 (2)	0.0346 (5)	
C1	0.7477 (2)	0.25092 (18)	0.6784 (2)	0.0334 (6)	
C2	0.8639 (2)	0.27954 (18)	0.6749 (2)	0.0387 (7)	
H2A	0.8848	0.2664	0.6104	0.046*	
C3	0.9458 (2)	0.32637 (19)	0.7655 (3)	0.0430 (7)	
C4	0.9165 (3)	0.3470 (2)	0.8639 (3)	0.0519 (8)	
H4A	0.9732	0.3785	0.9254	0.062*	
C5	0.8052 (3)	0.3209 (2)	0.8688 (3)	0.0453 (8)	
H5A	0.7853	0.3360	0.9331	0.054*	
C6	0.7184 (2)	0.27109 (18)	0.7778 (2)	0.0338 (6)	
C7	0.6055 (2)	0.24385 (18)	0.7899 (2)	0.0357 (6)	
H7A	0.5917	0.2612	0.8566	0.043*	
C8	0.4111 (2)	0.16962 (18)	0.7365 (2)	0.0357 (7)	
C9	0.3808 (3)	0.1909 (2)	0.8325 (3)	0.0446 (7)	
H9A	0.4343	0.2264	0.8899	0.053*	
C10	0.2727 (3)	0.1607 (2)	0.8449 (3)	0.0483 (7)	
C11	0.1927 (3)	0.1070 (2)	0.7575 (3)	0.0482 (8)	
C12	0.2238 (2)	0.0857 (2)	0.6625 (3)	0.0453 (7)	
H12A	0.1707	0.0501	0.6051	0.054*	
C13	0.3324 (2)	0.11598 (18)	0.6505 (3)	0.0363 (6)	
C14	0.3126 (2)	0.04987 (18)	0.4708 (3)	0.0400 (7)	
H14A	0.2437	0.0221	0.4762	0.048*	
C15	0.3415 (2)	0.03359 (18)	0.3702 (3)	0.0371 (6)	
C16	0.2637 (3)	−0.0209 (2)	0.2821 (3)	0.0495 (8)	
H16A	0.1975	−0.0472	0.2946	0.059*	
C17	0.2811 (3)	−0.0367 (2)	0.1799 (3)	0.0498 (8)	
H17A	0.2278	−0.0728	0.1234	0.060*	
C18	0.3811 (3)	0.0025 (2)	0.1622 (3)	0.0475 (8)	
C19	0.4612 (3)	0.0550 (2)	0.2466 (3)	0.0462 (7)	
H19A	0.5277	0.0800	0.2331	0.055*	
C20	0.4444 (2)	0.07135 (18)	0.3526 (2)	0.0354 (6)	
C21	1.0950 (3)	0.3371 (3)	0.6713 (3)	0.0656 (10)	
H21A	1.1776	0.3601	0.6857	0.098*	
H21B	1.0322	0.3587	0.5985	0.098*	
H21C	1.0990	0.2795	0.6644	0.098*	
C22	0.3392 (4)	−0.0663 (3)	−0.0224 (3)	0.0770 (12)	
H22A	0.3711	−0.0680	−0.0847	0.116*	
H22B	0.2497	−0.0522	−0.0560	0.116*	
H22C	0.3499	−0.1184	0.0151	0.116*	
C23	0.2426 (3)	0.1856 (3)	0.9503 (3)	0.0672 (11)	
H23A	0.3058	0.2237	0.9984	0.101*	
H23B	0.2439	0.1388	0.9975	0.101*	
H23C	0.1590	0.2100	0.9221	0.101*	
C24	0.0704 (3)	0.0742 (3)	0.7637 (4)	0.0730 (12)	
H24A	0.0351	0.0330	0.7041	0.110*	
H24B	0.0092	0.1172	0.7493	0.110*	
H24C	0.0895	0.0517	0.8416	0.110*	

### Atomic displacement parameters (Å^2^)

**Table:**

	*U*^11^	*U*^22^	*U*^33^	*U*^12^	*U*^13^	*U*^23^
Ni1	0.03026 (15)	0.0399 (2)	0.03049 (16)	−0.00596 (16)	0.01484 (12)	−0.00084 (19)
O1	0.0399 (9)	0.0571 (14)	0.0355 (11)	−0.0172 (9)	0.0211 (9)	−0.0088 (10)
O2	0.0435 (10)	0.0532 (13)	0.0317 (10)	−0.0148 (9)	0.0181 (9)	−0.0081 (10)
O3	0.0461 (11)	0.0767 (19)	0.0631 (14)	−0.0283 (11)	0.0289 (10)	−0.0166 (13)
O4	0.0989 (18)	0.078 (2)	0.0524 (15)	−0.0325 (15)	0.0394 (14)	−0.0308 (14)
N1	0.0332 (10)	0.0406 (14)	0.0335 (12)	−0.0018 (10)	0.0177 (10)	−0.0010 (11)
N2	0.0293 (10)	0.0376 (14)	0.0384 (13)	−0.0007 (9)	0.0150 (10)	0.0018 (11)
C1	0.0312 (12)	0.0367 (17)	0.0318 (14)	−0.0023 (11)	0.0122 (11)	0.0044 (12)
C2	0.0369 (13)	0.0477 (19)	0.0356 (15)	−0.0073 (12)	0.0185 (12)	−0.0032 (14)
C3	0.0364 (13)	0.047 (2)	0.0480 (17)	−0.0099 (12)	0.0192 (13)	−0.0003 (14)
C4	0.0483 (15)	0.064 (2)	0.0419 (16)	−0.0203 (16)	0.0158 (13)	−0.0151 (18)
C5	0.0468 (15)	0.056 (2)	0.0392 (16)	−0.0082 (13)	0.0231 (13)	−0.0091 (15)
C6	0.0329 (12)	0.0392 (17)	0.0305 (14)	−0.0028 (11)	0.0138 (11)	0.0018 (13)
C7	0.0395 (13)	0.0392 (17)	0.0344 (14)	0.0010 (12)	0.0209 (12)	−0.0004 (13)
C8	0.0338 (12)	0.0397 (19)	0.0392 (15)	−0.0021 (11)	0.0203 (12)	0.0029 (13)
C9	0.0430 (15)	0.0514 (19)	0.0482 (18)	−0.0059 (13)	0.0274 (14)	−0.0064 (15)
C10	0.0500 (15)	0.050 (2)	0.0586 (19)	0.0025 (15)	0.0364 (15)	0.0001 (18)
C11	0.0424 (15)	0.047 (2)	0.069 (2)	0.0002 (13)	0.0370 (16)	0.0050 (17)
C12	0.0353 (13)	0.0456 (19)	0.060 (2)	−0.0073 (13)	0.0243 (14)	−0.0015 (16)
C13	0.0322 (12)	0.0386 (17)	0.0430 (16)	0.0018 (11)	0.0201 (12)	0.0054 (14)
C14	0.0300 (12)	0.0404 (18)	0.0510 (17)	−0.0052 (12)	0.0176 (12)	−0.0004 (15)
C15	0.0337 (13)	0.0353 (17)	0.0409 (16)	0.0015 (11)	0.0135 (12)	−0.0026 (13)
C16	0.0381 (14)	0.048 (2)	0.060 (2)	−0.0085 (13)	0.0173 (15)	−0.0112 (17)
C17	0.0460 (16)	0.046 (2)	0.0474 (19)	−0.0041 (14)	0.0079 (14)	−0.0147 (16)
C18	0.0559 (17)	0.045 (2)	0.0410 (18)	−0.0002 (15)	0.0185 (15)	−0.0089 (15)
C19	0.0530 (16)	0.048 (2)	0.0401 (16)	−0.0102 (15)	0.0214 (14)	−0.0059 (15)
C20	0.0377 (13)	0.0348 (17)	0.0302 (14)	0.0000 (11)	0.0097 (12)	−0.0008 (12)
C21	0.0509 (16)	0.086 (3)	0.071 (2)	−0.0267 (19)	0.0368 (17)	−0.017 (2)
C22	0.094 (3)	0.081 (3)	0.053 (2)	−0.019 (2)	0.026 (2)	−0.029 (2)
C23	0.069 (2)	0.082 (3)	0.075 (3)	−0.0068 (19)	0.054 (2)	−0.009 (2)
C24	0.0556 (19)	0.082 (3)	0.103 (3)	−0.0189 (19)	0.055 (2)	−0.010 (2)

### Geometric parameters (Å, °)

**Table:**

Ni1—O2	1.8456 (19)	C10—C11	1.407 (5)
Ni1—O1	1.8485 (17)	C10—C23	1.508 (4)
Ni1—N2	1.856 (2)	C11—C12	1.380 (4)
Ni1—N1	1.861 (2)	C11—C24	1.517 (4)
O1—C1	1.305 (3)	C12—C13	1.388 (3)
O2—C20	1.306 (3)	C12—H12A	0.9300
O3—C3	1.367 (3)	C14—C15	1.411 (4)
O3—C21	1.425 (4)	C14—H14A	0.9300
O4—C18	1.358 (4)	C15—C20	1.411 (4)
O4—C22	1.422 (4)	C15—C16	1.412 (4)
N1—C7	1.301 (3)	C16—C17	1.358 (4)
N1—C8	1.428 (3)	C16—H16A	0.9300
N2—C14	1.304 (4)	C17—C18	1.395 (4)
N2—C13	1.417 (3)	C17—H17A	0.9300
C1—C6	1.414 (4)	C18—C19	1.376 (4)
C1—C2	1.416 (3)	C19—C20	1.402 (4)
C2—C3	1.367 (4)	C19—H19A	0.9300
C2—H2A	0.9300	C21—H21A	0.9600
C3—C4	1.404 (4)	C21—H21B	0.9600
C4—C5	1.356 (4)	C21—H21C	0.9600
C4—H4A	0.9300	C22—H22A	0.9600
C5—C6	1.417 (4)	C22—H22B	0.9600
C5—H5A	0.9300	C22—H22C	0.9600
C6—C7	1.417 (3)	C23—H23A	0.9600
C7—H7A	0.9300	C23—H23B	0.9600
C8—C9	1.386 (4)	C23—H23C	0.9600
C8—C13	1.393 (4)	C24—H24A	0.9600
C9—C10	1.384 (4)	C24—H24B	0.9600
C9—H9A	0.9300	C24—H24C	0.9600
			
Cg1···Cg2^i^	3.4737 (17)	Cg3···Cg3^i^	3.3760 (15)
Cg2···Cg3^i^	3.7196 (17)		
			
O2—Ni1—O1	83.33 (8)	C11—C12—H12A	119.1
O2—Ni1—N2	95.32 (9)	C13—C12—H12A	119.1
O1—Ni1—N2	178.38 (10)	C12—C13—C8	118.8 (3)
O2—Ni1—N1	177.06 (10)	C12—C13—N2	127.4 (3)
O1—Ni1—N1	95.26 (9)	C8—C13—N2	113.8 (2)
N2—Ni1—N1	86.13 (10)	N2—C14—C15	125.9 (2)
C1—O1—Ni1	127.25 (17)	N2—C14—H14A	117.1
C20—O2—Ni1	127.75 (17)	C15—C14—H14A	117.1
C3—O3—C21	117.9 (2)	C14—C15—C20	122.3 (2)
C18—O4—C22	118.7 (3)	C14—C15—C16	119.4 (3)
C7—N1—C8	121.1 (2)	C20—C15—C16	118.2 (3)
C7—N1—Ni1	125.95 (18)	C17—C16—C15	123.1 (3)
C8—N1—Ni1	112.92 (17)	C17—C16—H16A	118.5
C14—N2—C13	121.4 (2)	C15—C16—H16A	118.5
C14—N2—Ni1	125.10 (19)	C16—C17—C18	118.1 (3)
C13—N2—Ni1	113.45 (18)	C16—C17—H17A	121.0
O1—C1—C6	123.7 (2)	C18—C17—H17A	121.0
O1—C1—C2	117.5 (2)	O4—C18—C19	114.7 (3)
C6—C1—C2	118.8 (2)	O4—C18—C17	124.2 (3)
C3—C2—C1	120.5 (3)	C19—C18—C17	121.0 (3)
C3—C2—H2A	119.7	C18—C19—C20	121.2 (3)
C1—C2—H2A	119.7	C18—C19—H19A	119.4
C2—C3—O3	123.9 (3)	C20—C19—H19A	119.4
C2—C3—C4	120.8 (2)	O2—C20—C19	118.4 (3)
O3—C3—C4	115.3 (3)	O2—C20—C15	123.3 (2)
C5—C4—C3	119.8 (3)	C19—C20—C15	118.3 (3)
C5—C4—H4A	120.1	O3—C21—H21A	109.5
C3—C4—H4A	120.1	O3—C21—H21B	109.5
C4—C5—C6	121.4 (3)	H21A—C21—H21B	109.5
C4—C5—H5A	119.3	O3—C21—H21C	109.5
C6—C5—H5A	119.3	H21A—C21—H21C	109.5
C1—C6—C7	122.6 (2)	H21B—C21—H21C	109.5
C1—C6—C5	118.7 (2)	O4—C22—H22A	109.5
C7—C6—C5	118.7 (3)	O4—C22—H22B	109.5
N1—C7—C6	125.0 (3)	H22A—C22—H22B	109.5
N1—C7—H7A	117.5	O4—C22—H22C	109.5
C6—C7—H7A	117.5	H22A—C22—H22C	109.5
C9—C8—C13	119.8 (2)	H22B—C22—H22C	109.5
C9—C8—N1	126.5 (3)	C10—C23—H23A	109.5
C13—C8—N1	113.7 (2)	C10—C23—H23B	109.5
C10—C9—C8	121.6 (3)	H23A—C23—H23B	109.5
C10—C9—H9A	119.2	C10—C23—H23C	109.5
C8—C9—H9A	119.2	H23A—C23—H23C	109.5
C9—C10—C11	118.6 (3)	H23B—C23—H23C	109.5
C9—C10—C23	120.0 (3)	C11—C24—H24A	109.5
C11—C10—C23	121.3 (3)	C11—C24—H24B	109.5
C12—C11—C10	119.5 (3)	H24A—C24—H24B	109.5
C12—C11—C24	119.3 (3)	C11—C24—H24C	109.5
C10—C11—C24	121.1 (3)	H24A—C24—H24C	109.5
C11—C12—C13	121.7 (3)	H24B—C24—H24C	109.5
			
O2—Ni1—O1—C1	−177.0 (3)	C8—C9—C10—C11	0.1 (5)
N1—Ni1—O1—C1	5.6 (3)	C8—C9—C10—C23	−179.7 (3)
O1—Ni1—O2—C20	178.4 (2)	C9—C10—C11—C12	0.2 (5)
N2—Ni1—O2—C20	−0.7 (2)	C23—C10—C11—C12	−179.9 (3)
O1—Ni1—N1—C7	−1.8 (3)	C9—C10—C11—C24	−177.7 (3)
N2—Ni1—N1—C7	177.3 (3)	C23—C10—C11—C24	2.1 (5)
O1—Ni1—N1—C8	179.12 (19)	C10—C11—C12—C13	−0.1 (5)
N2—Ni1—N1—C8	−1.71 (19)	C24—C11—C12—C13	177.9 (3)
O2—Ni1—N2—C14	5.4 (2)	C11—C12—C13—C8	−0.4 (5)
N1—Ni1—N2—C14	−177.1 (2)	C11—C12—C13—N2	−178.7 (3)
O2—Ni1—N2—C13	−176.57 (19)	C9—C8—C13—C12	0.7 (4)
N1—Ni1—N2—C13	0.86 (19)	N1—C8—C13—C12	179.9 (2)
Ni1—O1—C1—C6	−6.3 (4)	C9—C8—C13—N2	179.3 (3)
Ni1—O1—C1—C2	174.40 (19)	N1—C8—C13—N2	−1.6 (4)
O1—C1—C2—C3	178.8 (3)	C14—N2—C13—C12	−3.3 (5)
C6—C1—C2—C3	−0.5 (4)	Ni1—N2—C13—C12	178.6 (2)
C1—C2—C3—O3	−179.9 (3)	C14—N2—C13—C8	178.3 (3)
C1—C2—C3—C4	0.0 (5)	Ni1—N2—C13—C8	0.2 (3)
C21—O3—C3—C2	−0.3 (5)	C13—N2—C14—C15	176.1 (3)
C21—O3—C3—C4	179.7 (3)	Ni1—N2—C14—C15	−6.0 (4)
C2—C3—C4—C5	−0.6 (5)	N2—C14—C15—C20	0.5 (5)
O3—C3—C4—C5	179.4 (3)	N2—C14—C15—C16	−178.0 (3)
C3—C4—C5—C6	1.7 (5)	C14—C15—C16—C17	176.6 (3)
O1—C1—C6—C7	1.9 (5)	C20—C15—C16—C17	−2.0 (5)
C2—C1—C6—C7	−178.8 (3)	C15—C16—C17—C18	0.4 (5)
O1—C1—C6—C5	−177.8 (3)	C22—O4—C18—C19	174.1 (3)
C2—C1—C6—C5	1.6 (4)	C22—O4—C18—C17	−5.2 (5)
C4—C5—C6—C1	−2.2 (5)	C16—C17—C18—O4	−179.8 (3)
C4—C5—C6—C7	178.2 (3)	C16—C17—C18—C19	1.0 (5)
C8—N1—C7—C6	177.6 (3)	O4—C18—C19—C20	−179.9 (3)
Ni1—N1—C7—C6	−1.4 (4)	C17—C18—C19—C20	−0.7 (5)
C1—C6—C7—N1	2.1 (5)	Ni1—O2—C20—C19	177.2 (2)
C5—C6—C7—N1	−178.3 (3)	Ni1—O2—C20—C15	−3.8 (4)
C7—N1—C8—C9	2.2 (5)	C18—C19—C20—O2	178.1 (3)
Ni1—N1—C8—C9	−178.7 (3)	C18—C19—C20—C15	−1.0 (5)
C7—N1—C8—C13	−176.9 (3)	C14—C15—C20—O2	4.7 (4)
Ni1—N1—C8—C13	2.2 (3)	C16—C15—C20—O2	−176.8 (3)
C13—C8—C9—C10	−0.6 (5)	C14—C15—C20—C19	−176.3 (3)
N1—C8—C9—C10	−179.6 (3)	C16—C15—C20—C19	2.2 (4)

Symmetry codes: (i) −*x*+1, −*y*, −*z*+1.

### Hydrogen-bond geometry (Å, °)

**Table:**

*D*—H···*A*	*D*—H	H···*A*	*D*···*A*	*D*—H···*A*
C7—H7A···O2^ii^	0.93	2.41	3.173 (3)	140

Symmetry codes: (ii) *x*, −*y*+1/2, *z*+1/2.
